# A Pilot Study of the Reliability and Agreement of Heart Rate, Respiratory Rate and Short-Term Heart Rate Variability in Elite Modern Pentathlon Athletes

**DOI:** 10.3390/diagnostics10100833

**Published:** 2020-10-16

**Authors:** Bartosz Hoffmann, Andrew A. Flatt, Luiz Eduardo Virgilio Silva, Marcel Młyńczak, Rafał Baranowski, Ewelina Dziedzic, Bożena Werner, Jakub S. Gąsior

**Affiliations:** 1 Physiotherapy Division, Faculty of Medical Sciences, Medical University of Warsaw, 02-091 Warsaw, Poland; bartosz.hoffmann@icloud.com; 2 Biodynamics and Human Performance Center, Department of Health Sciences and Kinesiology, Georgia Southern University (Armstrong Campus), Savannah, GA 31419, USA; aflatt@georgiasouthern.edu; 3 Department of Internal Medicine of Ribeirão Preto Medical School, University of São Paulo, Ribeirão Preto 14049-900, SP, Brazil; luizeduardo@usp.br; 4 Faculty of Mechatronics, Institute of Metrology and Biomedical Engineering, Warsaw University of Technology, 02-525 Warsaw, Poland; marcel.mlynczak@pw.edu.pl; 5 Department of Heart Rhythm Disorders, National Institute of Cardiology, 04-628 Warsaw, Poland; rb@ikard.pl; 6 Medical Faculty, Lazarski University in Warsaw, 02-662 Warsaw, Poland; ewelinadziedzic82@gmail.com; 7 Department of Pediatric Cardiology and General Pediatrics, Medical University of Warsaw, 02-091 Warsaw, Poland; bozena.werner@wum.edu.pl

**Keywords:** heart rate, respiratory rate, heart rate variability, reliability, repeatability, modern penthatlon, athletes

## Abstract

Research on reliability of heart rate variability (HRV) parameters in athletes has received increasing attention. The aims of this study were to examine the inter-day reliability of short-term (5 min) and ultra-short-term (1 min) heart rate (HR), respiratory rate (RespRate) and HRV parameters, agreement between short-term and ultra-short-term parameters, and association between differences in HR, RespRate and HRV parameters in elite modern pentathletes. Electrocardiographic recordings were performed in stable measurement conditions with a week interval between tests. Relative reliability was evaluated by intra-class correlation coefficients, absolute reliability was evaluated by within-subject coefficient of variation, and agreement was evaluated using Bland–Altman (BA) plot with limits of agreement and defined a priori maximum acceptable difference. Short-term HR, RespRate, log transformed (ln) root mean square of successive normal-to-normal interval differences (lnRMSSD), ln high frequency (lnHF) and SD2/SD1 HRV indices and ultra-short-term HR, RespRate and lnRMSSD presented acceptable, satisfactory inter-day reliability. Although there were no significant differences between short-term and ultra-short-term HR, RespRate and lnRMSSD, no parameter showed acceptable differences with BA plots. Differences in time-domain and non-linear HRV parameters were more correlated with differences in HR than with differences in RespRate. Inverse results were observed for frequency-domain parameters. Short-term HR, RespRate, lnRMSSD, lnHF, and SD2/SD1 and ultra-short-term HR, RespRate and lnRMSSD could be used as reliable parameters in endurance athletes. However, practitioners should interpret changes in HRV parameters with regard to concomitant differences in HR and RespRate and caution should be taken before considering 5 min and 1 min parameters as interchangeable.

## 1. Introduction

Comprehensive monitoring of fitness and performance as well as accurate diagnosis of fatigue, non-functional overreaching, and overtraining states are crucial for optimizing training and reducing risk of injury in elite professional sport [1,2,3,4,5,6]. In this regard, sensitive, non-invasive, time-efficient and cost-effective testing methods and biomarkers encompassing a multidimensional approach are being sought by coaches, exercise scientists and sports physicians to improve the evaluation of athletes [4,7,8,9].

Over the past decade, parameters associated with autonomic nervous system (ANS) regulation such as heart rate (HR) and heart rate variability (HRV), measures assessed during the post-exercise recovery period, have received increasing interest for monitoring training status and cardiovascular fitness [2,4,5,6,10,11,12,13,14,15,16,17,18,19,20,21,22,23,24,25]. Nevertheless, contradictory findings related to methodological inconsistencies and partial misinterpretation of the results limit the widespread implementation of HR and HRV measures in the sports field [4,18,20].

From the perspective of coaches and sports professionals, it is important to evaluate training status in as many athletes as possible relatively quickly and frequently to distinguish intended (e.g., due to training) from unintended (measurement error) changes using reliable measurements and validated tools. This will ensure reproducible results and enable meaningful findings [26,27]. Parameters (or tools) are rated as useful or sensitive based on providing high reliability and low test–retest variation [4]. 

Previous studies on reliability of HRV measures in athletes focused mainly on looking for an ultra-shortened reliable user-friendly HRV parameter [22,25,28,29,30,31,32,33,34]. It was suggested that the root mean square of successive differences between adjacent normal RR intervals (RMSSD), or its log transformed version (lnRMSSD), calculated based on 60 sec recordings is reliable [30] and displays strong agreement with RMSSD criterion derived from 5 min recordings [28,31,32]. Moreover, RMSSD is suggested to be the most appropriate and attractive parameter for use in elite endurance and team sports athletes [2,15,18,22,28,30,31]. 

Apart from the cardiac measurements, the basic parameter reflecting respiration (another part of the cardiorespiratory fitness) is respiratory rate. The assessment of its reliability and agreement can also be considered relevant, as some moderate causal effects might also influence the measurements of cardiac parameters. 

A less-studied group of endurance athletes are elite modern pentathletes. The modern pentathlon is an Olympic sport that consists of five different modalities (fencing, freestyle swimming, equestrian show jumping, and a combination of pistol shooting and cross-country running). Events last up to 8 h in duration, making energy and physiological demands close to maximal [35,36,37,38]. To date, there are few studies addressing modern pentathlon athletes [35,36,37,38,39,40,41] and we have found no data within the literature on the reliability of HRV in this population. Establishing the typical variation in HRV among this population is necessary to aid coaches in detecting meaningful changes related to pentathletes’ training status.

Despite the large number of methodological papers published on HRV [42,43,44,45,46,47,48,49,50,51], many studies in this area failed to provide the necessary details concerning data acquisition and measurements, so the experimental design could not be replicated in laboratories, clinical settings and sports field. The lack of details concerning methodological aspects of the study significantly limits confidence in interpretation [52,53]. A recent paper on HRV reliability stated that still little is known about the reliability of baseline 5 min (short-term) measurements of HRV and studies continue to differ with respect to important methodological characteristics [54]. Therefore, the presented study has the following aims: (i) to assess the inter-day reliability of short-term (5 min) HR, respiratory rate and selected time-domain, frequency-domain, and non-linear HRV parameters; (ii) to assess the inter-day reliability of ultra-short-term (1 min) HR, respiratory rate and RMSSD (popular in sports field); (iii) to determine the agreement between short-term (5 min) and ultra-short-term (1 min) parameters, and (iv) to verify the correlations between differences in HR, respiratory rate and HRV parameters in stable conditions in elite modern pentathletes.

## 2. Materials and Methods

### 2.1. Participants

A total of 12 elite modern Caucasian pentathletes (8♂) living in Warsaw (Poland), aged 17–26 years, with professional careers ranging from 7 to 15 years, participated in the study. The study group included medalists of the World Championships (*n* = 6) and European Championships (*n* = 8). Inclusion criteria were: being an active athlete [55] currently in possession of the modern pentathlon license from the National Association; being in the pre-season period; acceptance and compliance with the measurement rules (details in Measurement protocol); absence of diseases and/or regular use of medications affecting the cardiopulmonary system and/or interfering with the ANS. The study was approved by the University Ethical Committee (SKE 01-01/2017, 7 March 2017, Warsaw, Poland) and followed the rules and principles of the Helsinki Declaration. All athletes were informed of the aims and risks involved with the protocol and subsequently provided written informed consent prior to data collection.

### 2.2. Measurement Protocol

Body mass status was measured using Body Mass Index (BMI) defined as body mass (kilograms) divided by the squared height (meters). A questionnaire that supports the collection and control of many confounding variables influencing HRV proposed by Laborde et al. [56] was used. The athletes were informed in detail about the objectives of the study and measurement protocol by telephone conversations 3 months before and 2 weeks before the measurements, and also by e-mail with instructions and study procedures. Briefly, participants were instructed to sleep normally (as usual in the 5 days before examination), refrain from physical activity the day before and on the day of study, eat a light breakfast, and use the toilet (if needed) on the day of study before examinations. The examinations were carried out at least 1 h after home breakfast and before lunch. Each athlete underwent two electrocardiography recordings (ECGs) with 7 day intervals between measurements. Athletes declared participation in one training per day (typical for the pre-season period) within the days between examinations. The first examination was denoted as “Test”, and the second one was denoted as “Retest.” Both the Test and Retest recordings were performed under the same conditions, i.e., in a quiet, bright university room, with stable temperature and humidity adjusted by the group of researchers (BH, JSG, AK).

### 2.3. Electrocardiography (ECG) Acquisition

Twelve-lead, 6-min ECGs were performed in a supine position between 8:00 AM to 12:00 PM. All ECGs (sampling frequency = 1000 Hz) were performed using a portable PC with an integrated software system (Custo cardio 100 12-channel PC ECG system; Custo med GmbH, Ottobrunn, Germany). The athletes were cabled by a same-sex researcher [54]. On both study days, in order to stabilize HR and respiratory rate, the participants were asked to lie in the supine position for 10 min [57] before the beginning of the appropriate ECGs (used to calculate HRV parameters). Athletes were encouraged to refrain from speaking and moving during the ECG examination. All ECGs were assessed by an experienced cardiologist (RB). Recordings of ECGs and respiratory rate were started at the same time.

### 2.4. Respiratory Rate

**Figure 1:**
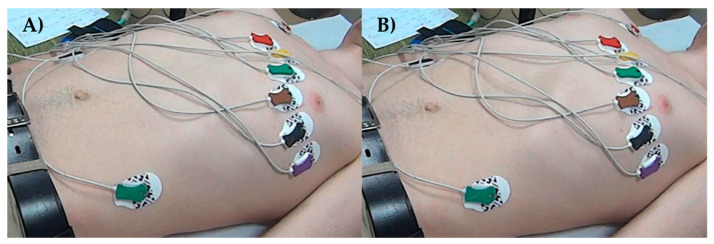
Picture from one of the videos, showing the athlete lying down: (**A**) end of inspiratory phase, (**B**) end of expiratory phase.

The athletes were not instructed how to breath during examinations (spontaneous breathing) to increase the applicability of the results in the sports field [11] but were informed that the breathing pattern would be video-recoded. Respiratory rate (RespRate) was monitored using the Sony^®^ HDRAS20 Action Camera with Wi-Fi. A picture from one video capture can be seen in Figure 1. Only the abdomen, thorax and neck were recorded. RespRate was determined from the counted number of respiratory cycles. The beginning of each respiratory cycle was defined as the end of the inspiratory phase when the diaphragm was at the apex. Calculation of the RespRate based on 5 min and 1 min video recordings (first minute of the 5 min recording) was independently performed by two researchers (BH, JSG). The disagreements between them were resolved through discussion.

### 2.5. HRV Analysis

The ECGs were visually inspected for potential non-sinus or aberrant beats and such erroneous beats were corrected from the cardiac interval series (RR series) before HRV analysis. The erroneous beats were manually corrected, i.e., one R-R interval before and one after each non-sinus beat were eliminated and replaced by R-R intervals computed by interpolation of degree zero based on the surrounding normal beats [58]. HR and HRV parameters were calculated based on appropriate ECGs (recordings started after stabilization period): (a) 5 min—criterion period (short-term parameters), and (b) 1 min ECGs (first minute of the 5 min recording—ultra-short-term parameters) using Kubios HRV Standard 3.4 software (University of Eastern Finland, Kuopio, Finland) [59,60].

Short-term time-domain, frequency-domain and nonlinear HRV parameters were calculated based on 5 min ECGs. Standard deviation of normal-to-normal RR intervals (SDNN), RMSSD, the log transformed RMSSD (lnRMSSD), log transformation of ratio between RMSSD (in ms) and mean RR interval (mRR, in ms), i.e., lnRMSSD/mRR and pNN50, which denotes the percent of RR intervals differing >50 ms from the preceding one, were determined. The following, popular in sport science, ultra-short-term parameters were calculated and analyzed based on 1 min ECGs: HR, RMSSD, (lnRMSSD) and lnRMSSD/mRR [2,15,18,21,22,28,30,31]. 

The usefulness of the frequency-domain HRV parameters for monitoring athletes in practice has been questioned. Some authors indicated several limitations of these parameters: (i) sensitivity to alterations in breathing rate and thus lower reliability [17]; (ii) analysis requires technical knowledge for interpretation; (iii) appropriate time needed for calculation may not be a suitable for analyzing athletes in a time-constrained setting [32]. Nevertheless, we decided to include the reliability assessment of the frequency-domain short-term (5 min) in the set of HRV parameters evaluated in this study. The recordings were obtained in stable conditions so that our findings could be considered as reference and prerequisites for future studies performed in the sports field. 

Before calculating spectral HRV parameters, smoothness priors based on the detrending approach was applied (smoothing parameter, Lambda value = 500) [61], and then RR interval series were transformed to an evenly sampled time series using a cubic spline interpolation followed by 4-Hz resampling. The detrended and interpolated RR interval series were used to compute HRV spectra by employing a fast-Fourier transform (FFT) with Welch’s periodogram method (300 s window width without overlap for 5 min ECGs). This definition ensures that the HRV spectral parameters were estimated from a single window, containing the whole 5 min period of recording. The range for respiratory rate was between 9 and 19 breaths/min in all subjects. Thus, the following bands for spectral components were securely distinguished: low-frequency (LF, 0.04–0.15 Hz) and high-frequency (HF, 0.15–0.40 Hz). The power at both bands were estimated in absolute (ms^2^) and normalized units (nu) [42]. Natural log transformed (ln) absolute powers in the LF (lnLF) and HF (lnHF) bands, the LF/HF ratio, and the powers in normalized units (nLF and nHF) were used for further analysis. From nonlinear HRV parameters, the ratio of Poincaré plot standard deviation along the line of identity to the standard deviation perpendicular to the line of identity (SD2/SD1), approximate entropy (ApEn), sample entropy (SampEn) and short-term fluctuations of detrended fluctuation analysis (DFAα1) were analyzed. Notice that HRV spectral and nonlinear parameters were obtained only for short-term (5 min) recordings.

### 2.6. Relationships between Differences in HR and RespRate and Differences in HRV Parameters

Most HRV studies have not accounted for the significant correlation between HRV parameters and mean HR [45,62,63,64]. This is an important consideration for sport practitioners and scientists using HRV to assess training status in athletes who typically present low resting HR [65,66]. Reduction in HRV, indicating, e.g., ANS stress, should be interpreted by taking into account respective changes in resting HR [67,68]. In addition to HR, we also assessed the correlation between differences in RespRate and differences in HRV.

### 2.7. Statistical Analysis

The Kolmogorov–Smirnov test was used to assess the normality of the data distribution. Natural log transformation (ln) was used if the data were not normally distributed. A paired Student′s *t*-test was employed to compare systematic changes between Test and Retest in analyzed parameters. Pearson’s correlation coefficient (r) was calculated to illustrate the relationship among differences (values from Retest—values from Test) in HR (HR-diff), RespRate (RespRate-diff) and HRV parameters. Due to the low sample size, the figures with correlations are more useful as illustrative than as analytic. The threshold probability of *p* < 0.05 was taken as the level of significance for all statistical tests. All calculations were performed using the STATISTICA 12 (StatSoft Inc., Tulsa, OK, USA) and MedCalc software version 19.4.1 (MedCalc Software, Ostend, Belgium). The Bland–Altman plots were created using MedCalc software version 19.4.1 (MedCalc Software, Ostend, Belgium). GraphPad Prism 5 (GraphPad Software Inc., San Diego, CA, USA, 2005) was used to create correlation plots.

#### 2.7.1. Reliability Statistics

Inter-day reliability of all parameters was calculated using the intraclass correlation coefficient (ICC), the within-subject coefficient of variation (WSCV) and Cohen′s d. The relative reliability of HR, RespRate and HRV parameters was analyzed using the ICC [69]. A priori, an ICC value between 0 to 0.30 was considered small, 0.31 to 0.49 moderate, 0.50 to 0.69 large, 0.70 to 0.89 very large, and 0.90 to 1.00 nearly perfect [70]. The absolute reliability was analyzed using typical error of measurement (WSCV) [71,72,73,74]. The WSCV less than 10% was considered highly reliable. Cohen’s d was utilized to determine the effect size of the mean differences between Test and Retest [75] with thresholds considered a priori as trivial (<0.2), small (0.2–0.6), moderate (0.6–1.2), large (1.2–2.0) or very large (>2.0) [70]. In general, the combination of a trivial or small Cohen’s d, ICC > 0.85 and WSCV < 10% was considered as acceptable, with satisfactory reliability.

#### 2.7.2. Agreement Statistics

Agreement between short-term (5 min) and ultra-short-term (1 min) HR, RespRate and HRV parameters (from Test and Retest separately) was verified using a Bland–Altman plot with limits of agreement (LoA) [76,77,78,79,80] and ICC [81] with interpretation proposed by Hopkins et al. [70]. The smallest worthwhile change (SWC, calculated using formula 0.2 × Test-values standard deviation) [82] was used to define the maximum allowed difference between methods presented in Bland–Altman plots. Two methods are considered in agreement if the LoA do not exceed the maximum allowed difference between methods (SWC). 

## 3. Results

### 3.1. Participants

Results of four participants (out of 12) were excluded from the analysis due to the detection of cardiac abnormalities in the recorded ECG (prolonged QTc interval > 450 ms, n = 2; left bundle branch block, n = 2). Consequently, results of 8 male athletes were included in the statistical analysis. The mean (± SD) age, weight, height, BMI and duration of professional athletic career were: 21.7 years (±3.1), 75.9 kg (±9.5), 182.6 cm (±6.1), 22.7 kg/m^2^ (±2.3) and 10.8 years (±2.9). Athletes declared participating in 19 training sessions (±2) per week during the normal in-season time.

### 3.2. Reliability of Short-Term (5 min) HR, RespRate and HRV Parameters

Table 1 presents the results of reliability statistics for short-term (5 min) HR, RespRate and HRV parameters. There were no significant differences in all analyzed parameters between Test and Retest (*p*-values between 0.25 and 0.94). Relative and absolute reliability of HR, RespRate, and all time-domain HRV parameters (with one exception, pNN50) were considered nearly perfect (ICC between 0.96 and 0.99) and high (WSCV% between 1.4 and 7.5), respectively, with trivial effect size (Cohen′s d between −0.18 and 0.15). Effect size for frequency-domain parameters were trivial (nLF, lnHF, nHF, LF/HF) or small (lnLF). lnLF presented large, nLF, lnHF, nHF very large, and LF/HF nearly perfect relative reliability (ICC between 0.66 and 0.93). Absolute reliability was considered low for all frequency domain parameters (WSCV% between 10.4 and 55.2) except for lnHF, which presented high absolute reliability (WSCV% = 6.1). The nonlinear parameters presented large (ApEn and SampEn: ICC = 0.63 and 0.67), very large (DFAα1 and SD2/SD1: ICC = 0.77 and 0.87) relative reliability and high (SD2/SD1, ApEn and SampEn: WSCV% = 8.9, 6.1 and 9.1) or low (DFAα1, WSCV% = 17.2) absolute reliability with small (ApEn, DFAα1) and trivial (SD2/SD1 and SampEn) effect size.

### 3.3. Reliability of Ultra-Short-Term (1 min) HR, RespRate, RMSSD, lnRMSSD and lnRMSSD/mRR

Table 2 presents results of reliability statistics for ultra-short-term (1 min) HR, RespRate and RMSSD indexes. There were no significant differences in all analyzed parameters between Test and Retest (*p*-value between 0.18 and 0.80). HR and RespRate presented very large or nearly perfect relative reliability and high absolute reliability with trivial effect size. Relative and absolute reliability of lnRMMSD and lnRMSSD/mRR were considered very large and high, respectively, with a small effect size. 

### 3.4. Agreement between Short-Term and Ultra-Short-Term Parameters

**Figure 2:**
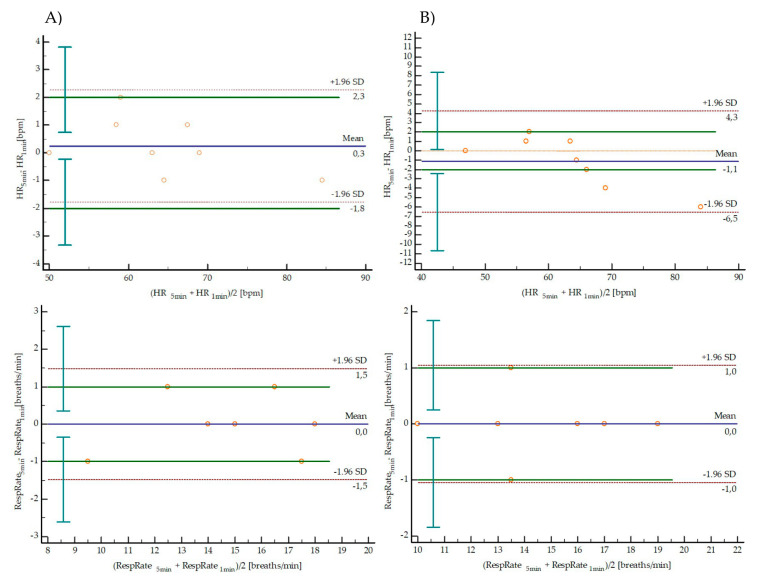
Bland–Altman plots representing the agreement between the 1 min and 5 min in HR and RespRate in both Test (column **A**) and Retest (column **B**) periods. The solid blue line indicates the bias, dotted red lines are the 95% LoA (±1.96 SD), vertical lines are confidence interval LoA limits, solid green lines are a priori defined maximum allowed difference.

**Figure 3:**
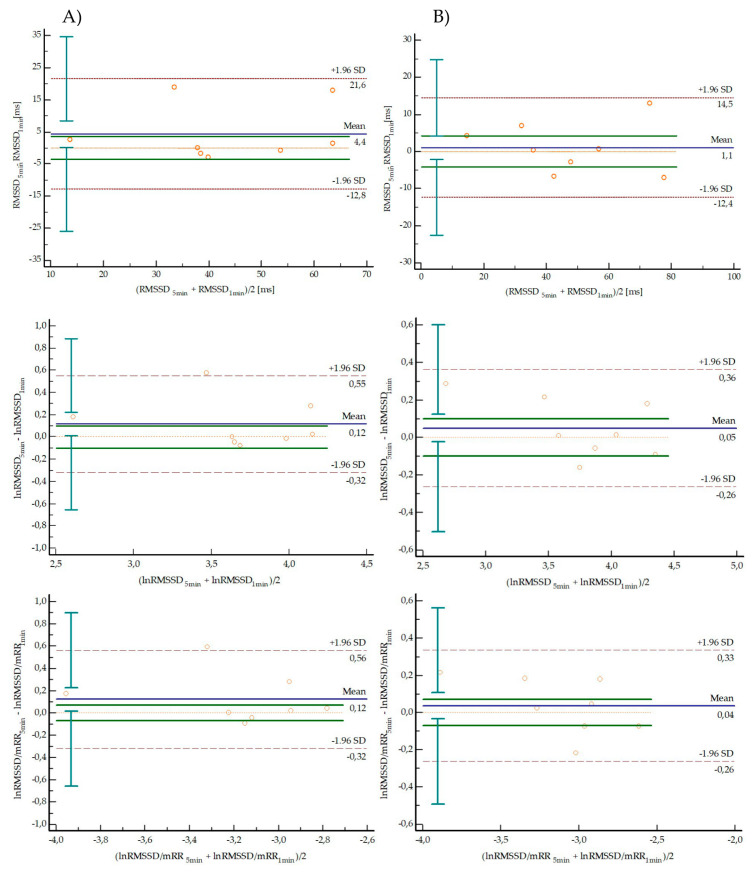
Bland–Altman plots representing the agreement between the 1 min and 5 min in selected HRV parameters in both Test (column **A**) and Retest (column **B**) periods. The solid blue line indicates the bias, dotted red lines are the 95% LoA (±1.96 SD), vertical lines are confidence interval LoA limits, solid green lines are a priori defined maximum allowed difference.

Table 3 and Figure 2 and Figure 3 present results of agreement between short-term (5 min) and ultra-short-term (1 min) HR, RespRate and selected HRV parameters from Test and Retest independently. There were no significant differences between short-term (5 min) and ultra-short-term (1 min) parameters in both Test and Retest. HR and RespRate presented nearly perfect agreement (ICC > 0.9) in Test and Retest with a trivial effect size. Small or trivial effect sizes of the mean difference between 5 min and 1 min parameters were observed for all measured time-domain indices in Test and Retest. RMSSD, lnRMSSD and lnRMSSD/mRR presented very large and nearly perfect agreement (ICC > 0.7) in Test and Retest. The Bland–Altman plots are shown in Figure 2 (HR and RespRate) and Figure 3 (RMSSD, lnRMSSD and lnRMSSD/mRR), representing the agreement between the 1 min and 5 min parameters in both Test (column A) and Retest (column B) periods. The LoA are defined as the mean difference ± 1.96 SD of differences. The 95% confidence intervals for upper and lower LoA are presented in Table 3. In all analyzed parameters, in both Test and Retest, LoA exceeded the defined maximum acceptable difference. 

### 3.5. Correlation between Differences in HR or RespRate and Differences in HRV Parameters

**Figure 4:**
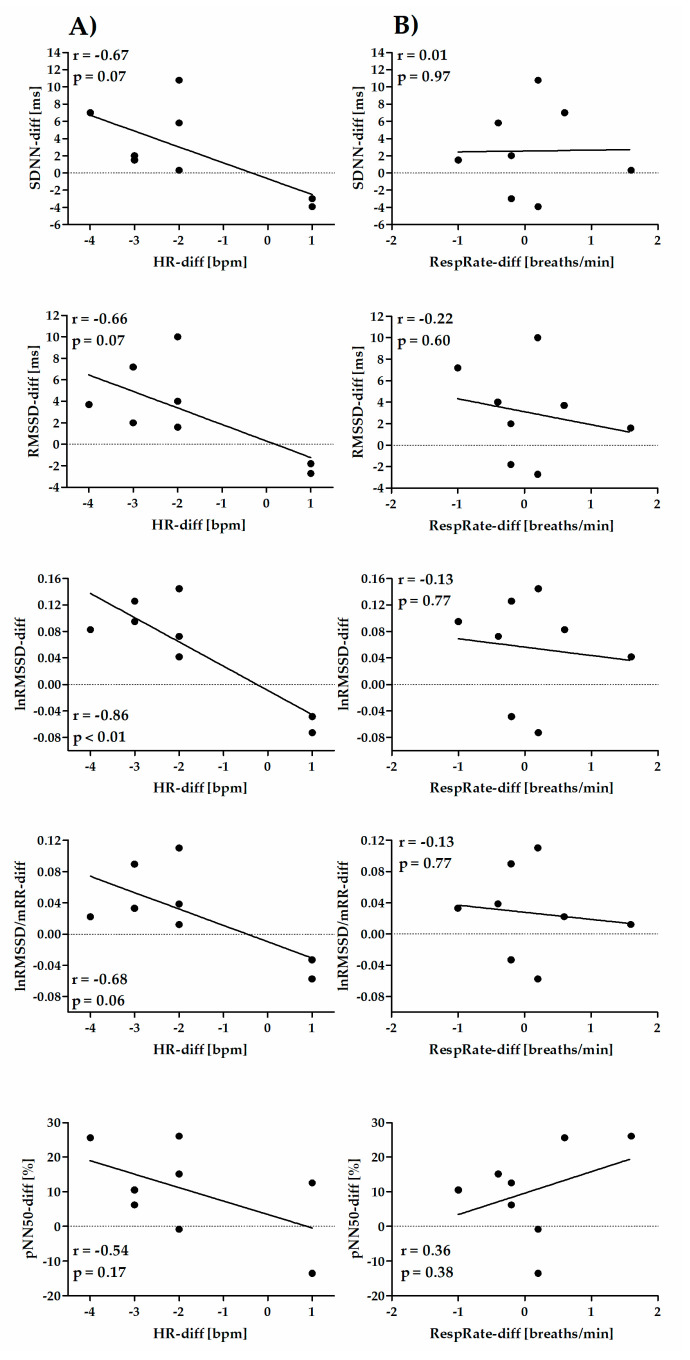
Pearson′s correlation coefficient between Retest–Test differences in time-domain short-term (5 min) HRV parameters and differences in HR (HR-diff) (column **A**) or RespRate (RespRate-diff) (column **B**).

**Figure 5:**
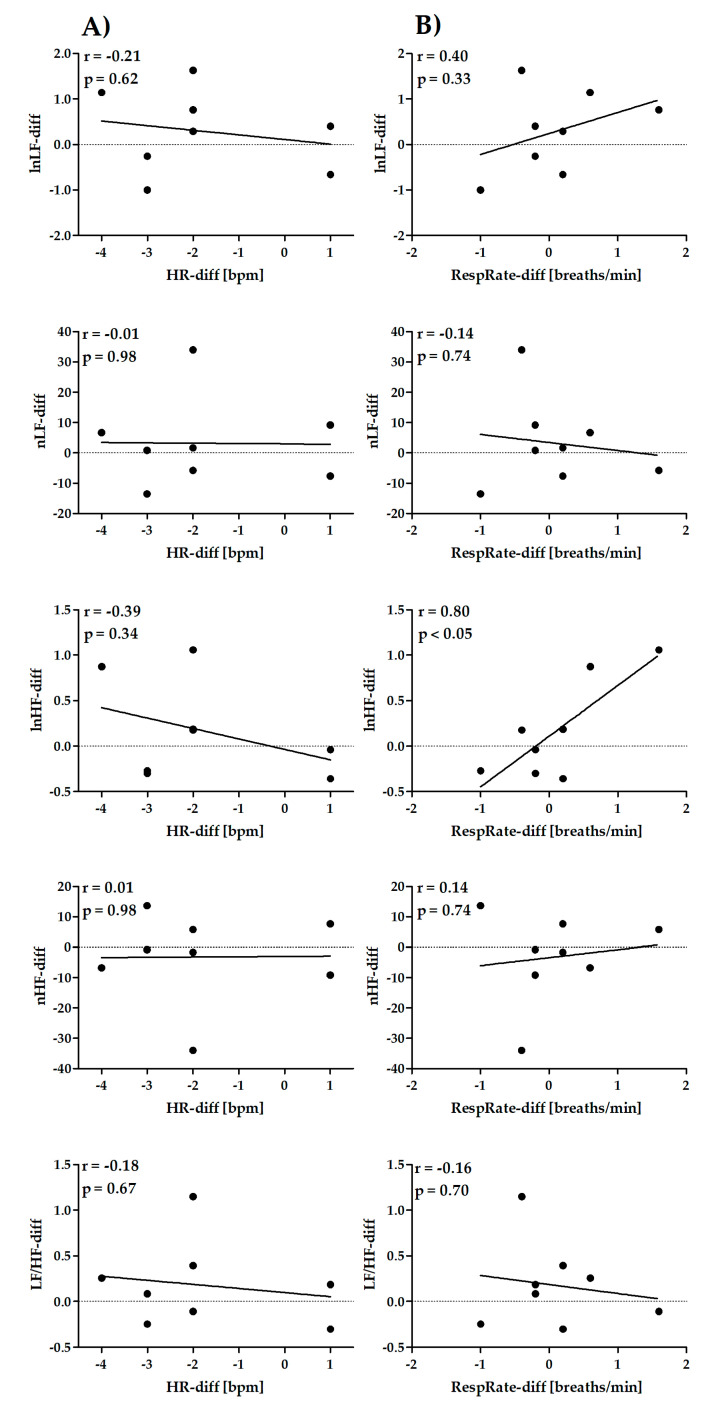
Pearson’s correlation coefficient between Retest–Test differences in frequency-domain short-term (5 min) HRV parameters and differences in HR (HR-diff) (column **A**) or RespRate (RespRate-diff) (column **B**).

**Figure 6:**
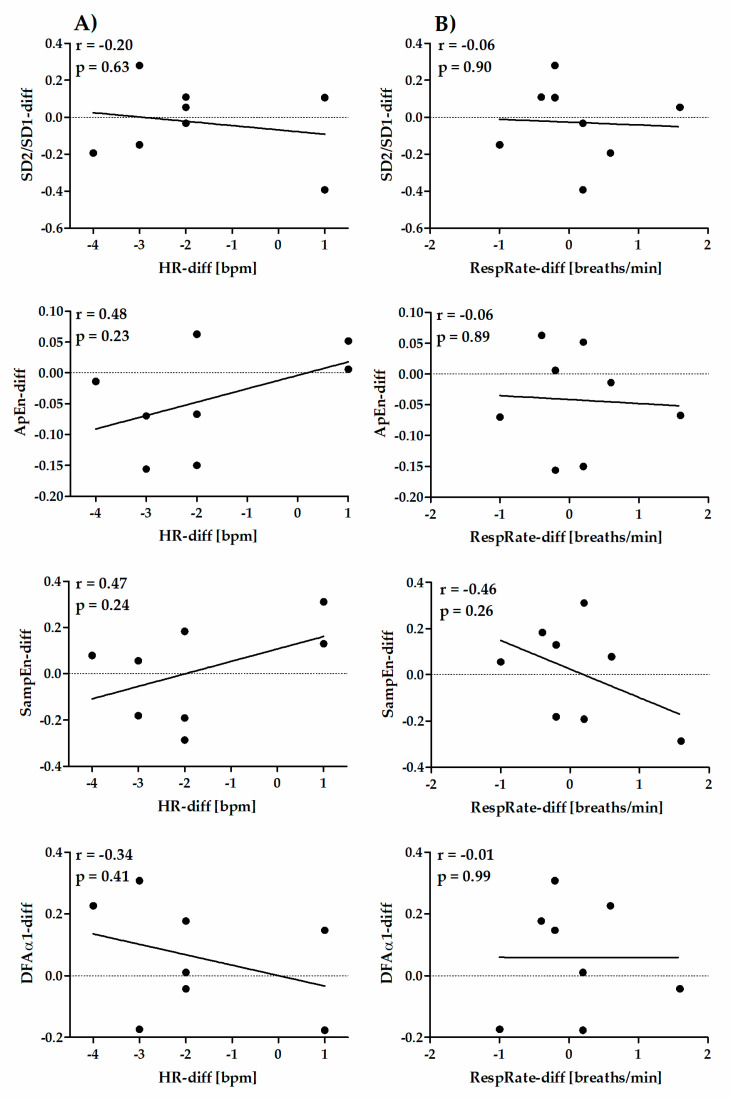
Pearson′s correlation coefficient between Retest–Test differences in nonlinear short-term (5 min) HRV indices and differences in HR (HR-diff) (column **A**) or RespRate (RespRate-diff) (column **B**).

**Figure 7:**
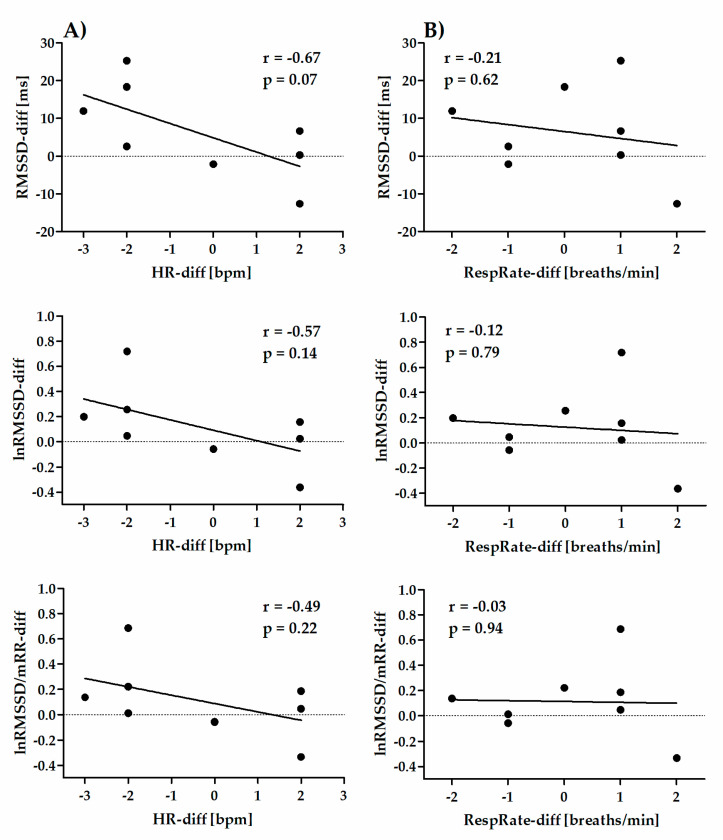
Pearson’s correlation coefficient between Retest–Test differences in ultra-short-term (1 min) HRV parameters and differences in ultra-short-term (1 min) HR (HR-diff) (column **A**) or ultra-short-term RespRate (RespRate-diff) (column **B**).

The correlations for Retest–Test differences between HRV parameters and HR (column A) or RespRate (column B) are shown in Figure 4 (time-domain short-term parameters), Figure 5 (frequency-domain short-term parameters), Figure 6 (nonlinear short-term parameters) and Figure 7 (ultra-short-term parameters). For short-term (5 min) time-domain and nonlinear HRV parameters, the Retest–Test differences are more correlated to the differences in HR (HR-diff) than to the differences in RespRate (RespRate-diff). Inversely, for short-term (5 min) frequency-domain HRV parameters (except for LF/HF), the Retest–Test differences are more correlated with the RespRate-diff than the HR-diff. Significant correlations were observed for the association between HR-diff and lnRMSSD-diff (r = −0.86, *p* < 0.01) and between RespRate-diff and lnHF-diff (r = 0.80, *p* < 0.05). For ultra-short-term (1 min) HRV parameters, the Retest–Test differences are more correlated (higher *r*) with HR-diff than with RespRate-diff.

## 4. Discussion

The purpose of this pilot study was to assess the reliability of short-term (5 min) and ultra-short-term (1 min) HRV parameters derived from ECG recordings performed in stable measurement conditions with one-week time interval between tests in elite modern pentathletes. Agreement between short-term and ultra-short-term parameters and correlation between differences in HR, RespRate and HRV parameters were also assessed. 

We showed that short-term (5 min) HR, RespRate, lnRMSSD (time-domain), lnHF (frequency-domain), SD2/SD1 (nonlinear) and ultra-short-term (1 min) HR, RespRate and lnRMSSD presented acceptable, satisfactory reliability. These results indicate that the aforementioned HRV parameters could be used by coaches and researchers as reliable parameters in endurance athletes during baseline periods in laboratory-controlled settings. Methodological differences between published studies, and general lack of studies on test-retest reliability analysis of HR, RespRate, short-term (5 min) and ultra-short-term (1 min) HRV parameters in elite athletes hinder comparisons. The current findings are somewhat in line with previous studies showing that time-domain indices are more reliable than spectral-domain parameters in moderately-trained males [11] and that ultra-short-term (1 min) lnRMSSD demonstrated acceptable interday reliability (ICC = 0.90, CV < 7%) in elite rugby union players [30]. 

Recently, many authors have adopted ultra-shortened, user-friendly HRV parameters for the evaluation of endurance and team-sport athletes; however, the analysis of the relevance of such parameters focused more on agreement with 5 min criterion parameters than on test-retest reliability [22,25,28,30,31,32,33,34]. Ultra-short-term (1 min) lnRMSSD has been shown to present strong agreement with criterion 5 min recordings [28,31]. No significant differences between short-term (5 min) and ultra-short-term (1 min) HR, RespRate and lnRMSSD were observed in the current study, which is somewhat in line with previous studies with endurance and team-sport athletes [22,28,31,32]. 

However, in our study, all analyzed ultra-short-term parameters, in both Test and Retest, showed a LoA with short-term parameters that exceeded the defined a priori maximum acceptable difference (SWC). This is contradictory to the high ICC values obtained between 5 min and 1 min HRV parameters. In studies evaluating agreement between short-term and ultra-short-term parameters, authors have not provided the maximum acceptable difference, nor did they report whether it was exceeded by the LoA. Therefore, the criteria for defining strong agreement in these studies are not well defined. Recent reviews have highlighted that important data from the Bland–Altman method are often omitted [79,80]. Abu-Arafeh et al. recently provided a comprehensive list of key items for reporting Bland–Altman analysis [79]. The first key item, not reported in published studies on agreement between ultra-short-term and criterion HRV parameters in athletes, is the definition of the a priori acceptable LoA, to define the minimal agreement needed to consider the new measurement method as interchangeable with another method (often gold standard or criterion method) [79]. lnRMSSD justifiably seems to be the preferred HRV index for monitoring athletic performance and training status due to several advantages described previously [2,15,18,21,22,28,30,31]. We confirm that ultra-short-term (1 min) lnRMSSD, in combination with HR and respiratory rate, can be reliably used in elite modern pentathletes. However, without defining a priori the maximum acceptable difference between 5 min and 1 min parameters, caution should be taken before considering these two methods as interchangeable, at least in this group of participants. From the results of our study, no parameter can be considered interchangeable for 1 min and 5 min, once the LoA between them exceeds the SWC. 

On the other hand, although the consideration of LoA < SWC represents an important criterion for agreement analysis, we believe it has an important limitation. Since LoA is calculated from the SD of differences between “Test” and “Retest”, LoA will be low whenever the differences from all subjects tend to be homogeneous. This does not happen only when the results from “Test” and “Retest” are the same, but also when they vary by the same amount in all individuals. In other words, the fixed bias is not taken into account in this comparison. We suggest that a more complete analysis would involve the comparison of LoA and SWC together with the one-sample *t*-test to check if the fixed bias is different from zero. However, since in our study no parameter presented a LoA wider than SWC, the one-sample *t*-test was needless.

In experimental conditions similar to those of the current study, the reliability of time-domain and frequency-domain HRV parameters was shown to be affected by differences in HR and RespRate in young healthy volunteers. HR was a stronger determinant for HRV reproducibility than RespRate, and even a minimal change of HR considerably altered HRV [83]. In elite athletes, the differences in short-term time-domain and nonlinear HRV parameters, as well as in ultra-short-term HRV parameters, turned out to be more correlated with differences in HR than with differences in RespRate. This supports the notion that changes in HRV indices should be assessed and interpreted with regard to concomitant changes in resting HR [4,15,18]. The normalization of HRV parameters concerning the HR level has already been suggested and we reinforce its importance for comparisons of people with different levels of HR [67,68]. 

Contrastingly, differences in short-term frequency-domain HRV parameters were more correlated with differences in RespRate than with differences in HR. Indeed, HRV (mostly frequency-domain parameters) is also highly affected by respiratory depth and rate [44,84]. Spectral powers at LF and HF bands are commonly attributed to sympathetic and vagal, and vagal influences alone on HR, respectively. Thus, coaches and sport practitioners should be aware that changes in the respiratory rate may confound spectral indices [53]. It has been suggested that depth of breathing could be even more important than its rate [5]. Młyńczak and Krysztofiak proposed cardiorespiratory temporal causal links with the path for lying supine from tidal volume, through heart activity variation and average heart activity, to respiratory timing [5]. Even if those links appeared rather moderate, the possible differences in the respiratory pattern should be taken into account in the protocol assessing the cardiac parameters. The measurement of tidal volumes using, e.g., mouthpieces may affect HRV data [17,85]. Moreover, there is no optimal solution on how to record and control respiratory depth and rate in HRV studies [44,53]. One possible solution for future studies in athletes could be to implement the Pneumonitor 2 or 3, a portable device that would register respiratory pattern (both depth and rate) together with single-lead ECG (enough to estimate aforementioned parameters), motion, and/or pulse oximetry (saturation, pulse wave) designed for environmental physiology analyses and sports medicine [86,87].

Although HRV parameters are often used as biomarkers of physical conditioning, as well as indicators of the severity of diseases, the physiological meaning of such parameters are not well understood in many situations. While the spectral power at the HF band is widely accepted to represent the vagal modulation (respiratory sinus arrhythmia) to the heart under normal respiratory frequencies (>9 breaths/min), the physiological interpretation of other indices is not so clear. This is the case of most nonlinear HRV parameters. For example, the short-term scaling exponent (DFAα1) represents the fractal correlations present in RR series and was demonstrated to represent one of the best risk factors for patients with heart failure or myocardial infarction [88,89]. However, the changes in fractal structure of RR series in these patients are likely to be the consequence of the global change in cardiovascular function, and not a marker of any specific physiological variable. The living organism can be considered a complex system and, as such, it is difficult to disentangle the influences caused by each mechanism, once their functions are highly interdependent [90]. This is the reason why many nonlinear HRV parameters are considered as “complexity measures”, in the sense that, although they are not clearly associated with specific physiological meanings, they are able to represent the general complexity of the system, attested by their important role as risk factors, prognosis and fitness [91,92].

No previous study on inter-day reliability of HRV in athletes with similar methodological characteristics to our study was found. As underlined by many authors, there are still substantial methodological inconsistencies throughout the literature regarding HRV in sports science that limit comparison [4,18,93]. Moreover, the authors of a recent study on test-retest reliability of short-term HRV parameters suggested that the studies differ with respect to important methodological issues and evidence on this topic is far from clear [54]. There are several methodological characteristics that may cause differences in findings among studies on HRV reliability, e.g., reliability statistics adopted, number of tests performed and the time interval between them, and sample heterogeneity [54]. Lack of information about other methodological aspects also limits potential for study replication and confidence in interpretation [52,53], such as the lack of information on ECG acquisition and processing, including device, software, recording duration and conditions, breathing control, and position during recordings. Thus, we emphasize the importance of providing all the necessary information for reproducibility, as well as the standardization of statistics for the analysis of reliability of biomarker parameters in athletes.

Our pilot study was limited by a small homogenous sample size taken at a given moment of the sport season, which limits generalizability of the study’s findings. The consequence of low sample size is the illustrative character of the correlation data. HRV data were obtained during supine ECG recordings in controlled laboratory settings. Heart rate monitors or smartphone with HRV application may be more useful to collect RR intervals in athletes than traditional ECG, especially outside of a laboratory setting [94,95,96]. Nevertheless, as suggested by Lucini et al., caution must be applied when assessing HRV using devices that cannot discriminate RR series between sinus and non-sinus beats, especially in athletes [57]. In our study, four participants were excluded from the analysis due to presence of cardiac abnormalities. Recently, abnormal ECG changes were observed in about 4% of top-level endurance athletes [97]. Therefore, we suggest performing ECG screening among examined athletes before including the data for detailed HRV analysis.

## 5. Conclusions

Short-term HR, RespRate, short-term lnRMSSD, lnHF, and SD2/SD1 HRV parameters and ultra-short-term HR, RespRate and lnRMSSD could be used by coaches, sport practitioners and researchers as reliable parameters in elite modern pentathlon athletes at baseline examinations in laboratory settings. Without defining the a priori maximum acceptable difference, caution should be taken before considering HR, RespRate and lnRMSSD from 5 min and 1 min recordings as interchangeable. Moreover, differences in short-term and ultra-short-term HRV parameters should be assessed considering the concomitant differences in HR and RespRate.

## Figures and Tables

**Table 1:** Reliability results for HR, RespRate and selected short-term 5 min HRV parameters.

	Test Mean ± SD	Retest Mean ± SD	*p*	Cohen′s d (95% CI)	ICC (95% CI)	WSCV% (95% CI)
HR [bpm]	64.6 ± 9.8	62.9 ± 9.7	0.73	−0.18 (−0.35–−0.04)	0.97 (0.71–0.99)	2.8 (1.2–4.6)
RespRate [breaths/min]	15 ± 3	15 ± 3	0.94	0.04 (−0.18–0.33)	0.96 (0.84–0.99)	3.4 (1.4–5.4)
SDNN [ms]	43.3 ± 16.6	45.9 ± 20.0	0.78	0.14 (−0.11–0.36)	0.96 (0.81–0.99)	7.5 (3.0–12.2)
RMSSD [ms]	45.2 ± 17.9	48.2 ± 21.1	0.76	0.15 (0.01–0.33)	0.97 (0.81–0.99)	6.7 (2.7–10.9)
lnRMSSD	3.72 ± 0.48	3.78 ± 0.49	0.83	0.11 (−0.05–0.25)	0.98 (0.89–0.99)	1.8 (0.7–2.9)
lnRMSSD/mRR	−3.12 ± 0.35	−3.09 ± 0.35	0.88	0.08 (−0.08–0.20)	0.99 (0.94–1.00)	1.4 (0.6–2.2)
pNN50	21.9 ± 16.6	32.1 ±17.3	0.25	0.60 (−0.03–1.37)	0.61 (−0.02–0.90)	152.4 (46.0–336.2)
lnLF	6.22 ± 1.05	6.51 ± 1.11	0.60	0.27 (−0.50–1.24)	0.66 (0.03–0.92)	10.4 (4.1–17.1)
nLF [nu]	44.8 ± 20.4	48.0 ± 21.7	0.77	0.15 (−0.21–0.95)	0.78 (0.23–0.95)	31.2 (11.8–54.1)
lnHF	6.43 ± 0.94	6.60 ± 1.02	0.74	0.17 (−0.27–0.86)	0.86 (0.47–0.97)	6.1 (2.4–9.8)
nHF [nu]	55.2 ± 20.3	52.0 ± 21.7	0.77	−0.15 (−0.95–0.21)	0.78 (0.23–0.95)	18.9 (7.4–31.8)
LF/HF	1.17 ± 1.17	1.35 ± 1.28	0.78	0.15 (−0.10–0.89)	0.93 (0.71–0.98)	55.2 (19.7–101.3)
SD2/SD1	1.69 ± 0.37	1.66 ± 0.42	0.89	−0.07 (−0.55–0.37)	0.87 (0.48–0.97)	8.9 (3.5–14.5)
ApEn	1.09 ± 0.11	1.05 ± 0.09	0.42	−0.42 (−0.91–0.33)	0.63 (0.02–0.91)	6.1 (2.5–9.9)
SampEn	1.67 ± 0.20	1.69 ± 0.29	0.92	0.05 (−0.63–0.74)	0.67 (−0.05–0.93)	9.1 (3.6–14.9)
DFAα1	0.87 ± 0.23	0.93 ± 0.31	0.67	0.23 (−0.34–0.82)	0.77 (0.26–0.95)	17.2 (6.7–28.7)

HR—Heart rate, RespRate—respiratory rate, HRV—Heart rate variability, SDNN—Standard deviation of the normal-to-normal RR intervals, RMSSD—Root mean square of successive RR interval differences, mRR—Mean RR interval, ln—Natural log transformed, pNN50—Percent of RR intervals differing >50 ms from the preceding one, LF—Low frequency, HF—High frequency, SD—Standard deviation, ApEn—Approximate entropy, SampEn—Sample entropy, DFAα1—Detrended fluctuation analysis (short-term fluctuations), ICC—Intra-class correlation coefficient, WSCV—Within-subject coefficient of variation, CI—confidence interval, bmp—Beats per minute, ms—Milliseconds, nu—Normalized units.

**Table 2:** Reliability results for ultra-short-term (1 min) HR, RespRate and RMSSD indexes.

	Test Mean ± SD	Retest Mean ± SD	*p*	Cohen’s d	ICC (95% CI)	WSCV% (95% CI)
HR [bpm]	64.4 ± 10.3	64.0 ± 11.9	0.63	−0.03 (−0.24–0.15)	0.98 (0.92–0.99)	2.5 (1.0–3.9)
RespRate [breaths/min]	14.6 ± 2.8	14.8 ± 2.8	0.80	0.05 (−0.36–0.46)	0.89 (0.56–0.98)	6.3 (2.5–10.2)
RMSSD [ms]	40.8 ± 16.7	47.1 ± 21.7	0.18	0.33 (−0.15–0.86)	0.78 (0.29–0.95)	24.8 (9.5–42.2)
lnRMSSD	3.61 ± 0.53	3.73 ± 0.58	0.30	0.22 (−0.21–0.85)	0.84 (0.45–0.97)	6.3 (2.5–10.2)
lnRMSSD/mRR	−3.24 ± 0.40	−3.13 ± 0.44	0.31	0.27 (−0.26–1.01)	0.75 (0.23–0.94)	6.7 (2.7–10.9)

HR—heart rate, RespRate—respiratory rate, RMSSD—Root mean square of successive RR interval differences, mRR—Mean RR interval, ln—Natural log transformed, ICC—Intra-class correlation coefficient, WSCV—Within-subject coefficient of variation, CI—Confidence interval, bmp—Beats per minute, ms—Milliseconds, SD—Standard deviation.

**Table 3:** Results of agreement between selected short-term (5 min) and ultra-short-term (1 min) parameters in Test and Retest.

	Parameter	Mean ± SD 5 min	Mean ± SD 1 min	*p*	Mean Difference (95% CI)	SWC	LoA	95% CI for Lower; Upper LoA	ICC (95% CI)
Test	HR [bpm]	64.6 ± 9.8	64.4 ± 10.3	0.52	0.2 (−0.6–1.1)	2.0	−1.8–2.3	−3.3–−0.2; 0.7–3.8	0.99 (0.98–0.99)
	RespRate [breaths/min]	15 ± 3	15 ± 3	0.50	0.0 (−0.6–0.6)	1.0	−1.5–1.5	−2.6–−0.4; 0.4–2.6	0.97 (0.85–0.99)
	RMSSD [ms]	45.2 ± 17.9	40.8 ± 16.7	0.20	4.4 (−2.9–11.7)	3.6	−12.8–21.6	−25.9–0.3; 8.5–34.7	0.86 (0.48–0.97)
	lnRMSSD	3.72 ± 0.48	3.61 ± 0.53	0.18	0.12 (−0.07–0.30)	0.10	−0.32–0.55	−0.65–0.01; 0.22–0.88	0.89 (0.58–0.98)
	lnRMSSD/mRR	−3.12 ± 0.35	−3.24 ± 0.40	0.17	0.12 (−0.07–0.31)	0.07	−0.32–0.56	−0.66–0.02; 0.23–0.90	0.80 (0.32–0.96)
Retest	HR [bpm]	62.9 ± 9.7	64.0 ± 11.9	0.29	−1.1 (−3.4–1.2)	2.0	−6.5–4.3	−10.6–−2.4; 0.2–8.4	0.97 (0.86–0.99)
	RespRate [breaths/min]	15 ± 3	15 ± 3	0.36	0.0 (−0.5–0.5)	1.0	−1.1–1.1	−1.9–−0.3; 0.3–1.9	0.98 (0.92–0.99)
	RMSSD [ms]	48.2 ± 21.1	47.1 ± 21.7	0.68	1.1 (−4.7–6.8)	4.2	−12.4–14.5	−22.6–−2.1; 4.2–24.7	0.95 (0.79–0.99)
	lnRMSSD	3.78 ± 0.49	3.73 ± 0.58	0.42	0.05 (−0.09–0.18)	0.10	−0.26–0.36	−0.50–−0.03; 0.12–0.60	0.96 (0.82–0.99)
	lnRMSSD/mRR	−3.09 ± 0.35	−3.13 ± 0.44	0.53	0.04 (−0.09–0.16)	0.07	−0.26–0.33	−0.49–−0.04; 0.11–0.56	0.93 (0.71–0.99)

HR—Heart rate, RespRate—Respiratory rate, RMSSD—Root mean square of successive RR interval differences, mRR—Mean RR interval, ln—Natural log transformed, ICC—Intra-class correlation coefficient, SWC—Smallest worthwhile change, LoA—Limits of agreement, CI—Confidence interval, bmp–Beats per minute, ms—Milliseconds, SD—Standard deviation.
